# Mis-spliced *FMR1* transcripts in human fragile X syndrome neural progenitors and neurons

**DOI:** 10.1186/s11689-026-09686-0

**Published:** 2026-04-02

**Authors:** Shaima M. Hourani, Kagistia Hana Utami, Sher Li Oh, Maija L. Castrén, Mahmoud A. Pouladi

**Affiliations:** 1 Department of Medical Genetics, Centre for Molecular Medicine and Therapeutics, Djavad Mowafaghian Centre for Brain Health, Edwin S. H. Leong Centre for Healthy Aging, Faculty of Medicine, British Columbia Children’s Hospital Research Institute, University of British Columbia, 950 West 28th Avenue, Vancouver, BC V5Z 4H4 Canada; 2 Duke NUS Medical School, Singapore, Singapore; 3 Department of Physiology, Faculty of Medicine, University of Helsinki, Helsinki, Finland

**Keywords:** Fragile X syndrome, *FMR1* mis-splicing, RNA splicing, *FMR1* reactivation, 5AzadC, Human neurons, Patient derived iPSCs, RNA-sequencing

## Abstract

**Background:**

Fragile X syndrome (FXS) is a neurodevelopmental disorder caused by loss of fragile X messenger ribonucleoprotein (FMRP). In most cases, this results from a CGG expansion exceeding 200 repeats in the 5’ untranslated region of the *fragile X messenger ribonucleoprotein 1* (*FMR1*) gene, known as the “full mutation”. While the trinucleotide expansion has long been thought to induce epigenetic silencing of this locus, studies have shown that many males with a full mutation still express *FMR1* mRNA. However, these individuals produce little to no FMRP protein, due to mechanisms that remain unclear. Mis-splicing of *FMR1* transcripts with an expanded CGG tract has recently been proposed as a potential mechanism underlying the absence of FMRP in FXS tissues despite the presence of gene transcripts.

**Methods:**

We used human neural progenitors and neurons differentiated from FXS human pluripotent stem cells and RNA-seq to examine splicing patterns of expanded *FMR1* transcripts. We analyzed transcript structure and protein expression to validate mis-splicing mechanisms.

**Results:**

We demonstrate an enrichment in levels of mis-spliced transcripts in human neural progenitors and neurons differentiated from FXS human pluripotent stem cells. Our findings confirm that expanded transcripts undergo aberrant splicing, which may contribute to the absence of FMRP despite transcriptional activity. We further show that pharmacological reactivation of the *FMR1 *locus results in expression of mis-spliced transcripts and that FMRP loss alone, in the absence of an expanded CGG tract, causes only a modest increase in splicing defect.

**Conclusions:**

These results suggest that mis-splicing may represent one of several mechanisms contributing to the absence of FMRP in FXS, specifically in cases where transcriptional silencing of the *FMR1* locus is incomplete and expanded transcripts are still produced. In these cases, expanded *FMR1* transcripts are produced but undergo aberrant processing that prevents functional protein production. These findings have important implications for understanding FXS pathogenesis and developing therapeutic strategies targeting the expanded locus.

**Supplementary Information:**

The online version contains supplementary material available at 10.1186/s11689-026-09686-0.

## Background

Fragile X syndrome (FXS) is the most common inherited cause of intellectual disability and autism spectrum disorders, affecting approximately 1 in 4,000 males and 1 in 8,000 females. FXS is caused by disruption of the *Fragile X Messenger Ribonucleoprotein 1* (*FMR1*) gene, resulting in deficiency or absence of its protein product, FMR1 protein (FMRP) [1]. FMRP is an RNA-binding protein that plays a critical role in synaptic plasticity and neuronal development by regulating the translation of hundreds of target mRNAs at synapses [2]. In most individuals with FXS, FMRP loss stems from a trinucleotide repeat expansion within the 5’ untranslated region (UTR) of *FMR1*. In unaffected individuals, the CGG tract length in this polymorphic region ranges between 5 and 55 repeats. Expansions between 56 and 200 CGG repeats, termed the “premutation,” cause excessive *FMR1* expression and increase the risk of developing fragile X-associated tremor/ataxia syndrome (FXTAS) and fragile X-associated primary ovarian insufficiency (FXPOI) [3, 4]. In contrast, CGG expansions exceeding 200 repeats, termed the “full mutation,” lead to hypermethylation, heterochromatin formation, and epigenetic silencing of the *FMR1* promoter. While this has traditionally been thought to result in complete absence of FMRP, 12–41% of males with FXS exhibit residual FMRP expression [3]. This residual expression has been attributed to intercellular mosaicism in CGG repeat size or methylation status, the latter being referred to as “partially methylated full mutations” [1, 3, 5].

Previous studies have shown that many males with methylated full mutations do express *FMR1* mRNA but still exhibit little to no FMRP and present phenotypes similar to those with no *FMR1* expression [6–9]. Several mechanisms have been proposed to contribute to the inhibition of *FMR1* mRNA translation in such individuals, including repeat-associated non-AUG-initiated (RAN) translation (CGG RAN) or the formation of extensive secondary structures by the expanded *FMR1* transcripts that impair ribosomal scanning [7, 8].

However, a recent study by Shah and colleagues proposed a novel mechanism underlying the reduction or absence of FMRP in individuals expressing expanded *FMR1* transcripts [10]. The authors detected *FMR1* RNA transcripts in leukocytes from FXS patients, some at levels comparable to typically developing individuals (TDI) due to mosaicism in CGG repeat number or partial methylation of full mutations. Importantly, mis-splicing of the *FMR1* transcript was proposed to account for the reduction or complete absence of FMRP in these individuals [10]. The mis-spliced isoform, *FMR1-217*, contains two exons, with the second being a pseudo-exon formed by retention of the first *FMR1* intron. This 1.8 kb transcript is predicted to encode a truncated 31-amino acid polypeptide with unknown function [10].

In this study, we provide independent validation of the observation that expanded *FMR1* transcripts undergo mis-splicing, preventing FMRP production in FXS. Using RNA-seq, we demonstrate the presence of the *FMR1-217* isoform in neural progenitors (NPCs) and neurons derived from FXS embryonic stem cells (ESCs). We further provide insights by investigating whether *FMR1* transcripts expressed following pharmacological reactivation of silenced *FMR1* in patient-derived iPSCs undergo proper splicing and restoration of FMRP expression. Additionally, we establish for the first time that the splicing defect occurs predominantly independently of FMRP loss itself, revealing a direct consequence of the CGG expansion on transcript processing.

## Materials and methods

### Cell culture and hESC/hiPSC maintenance

The cell lines used in this study included control H1/WA01 (male) hESCs [11], isogenic H1 *FMR1*KO hESCs [12], and WCMC-37 FXS hESCs (male, approximately 450 CGG repeats with partial methylation of the *FMR1* promoter; [13, 14], as well as two previously described controls (HEL11.4 and HEL46.11) and FXS (HEL100.2) patient-derived hiPSC lines [15]. The H1 *FMR1*KO line was generated using CRISPR/Cas9-mediated targeting of exon 3 of *FMR1* in the H1 hESC line as described previously [12]. All hESC and hiPSC lines were maintained under feeder-free conditions on Geltrex (ThermoFisher, Cat. #A1413302)-coated plates in mTeSR1 medium (STEMCELL Technologies, Cat. #85,850) at 37 °C with 5% CO_2_. Medium was replaced every 2–3 days, and cells were passaged weekly by 6-min incubation with Versene (ThermoFisher, Cat. #15,040,066).

### Neural progenitor cell and neuronal differentiation

H1, H1 *FMR1*KO, and WCMC-37 FXS hESCs, as well as control (HEL11.4) and FXS (HEL100.2; male; > 200 CGG repeats, fully methylated) hiPSCs, were differentiated into neural progenitor cells (NPCs) and neurons according to previously published protocols [12].

For NPC induction, hESCs and iPSCs were single-cell dissociated and plated at a density of 30,000 cells/cm^2^ in neural induction media (NIM, DMEM/F12:NeuroBasal media 1:1 with 1% N2, 2% B27, 1% PenStrep, 1% GlutaMax, 10 ng/ml hLIF, and 5 μg/ml Bovine Serum Albumin) containing 4 μM CHIR99021 (Tocris), 3 μM SB431542 (Sigma), and 0.1 μM Compound E (Millipore) for seven days. The culture was then split at a 1:3 ratio for the next five passages using Accutase in NIM without Compound E on Matrigel-coated plates.

For neuronal differentiation, NPCs were plated at a density of 20,000 cells/cm^2^ on 50 μg/ml poly-L-ornithine/10 μg/ml laminin-coated plates and grown in NeuroDiff media (DMEM/F12/Neurobasal media (1:1) supplemented with 1% N2, 2% B27, 20 ng/ml GDNF (R&D Systems), 20 ng/ml BDNF (R&D Systems), 300 μM dibutyryl-cyclic AMP (D0260, Sigma Aldrich), and 200 nM L-Ascorbic Acid (A4403, Sigma Aldrich) for at least three weeks. Medium was changed every 2–3 days.

### Immunofluorescence staining

Cells were plated on ethanol-treated coverslips and fixed with 4% formaldehyde in phosphate-buffered saline (PBS) for 15 min at room temperature. Following fixation, cells were washed with Tris-Buffered Saline (TBS) and incubated at room temperature for 45 min in blocking buffer (TBS containing 5% goat serum, 1% Bovine Serum Albumin, and 0.1% Triton-X-100 [Sigma Aldrich]). Fixed cells were incubated with primary antibodies in blocking buffer without Triton-X-100 overnight at 4 °C. The primary antibodies used were: anti-MAP2 rabbit polyclonal (AB5622, EMD Millipore), anti-TUJ1 mouse monoclonal (MAB1637, Merck-Millipore), anti-TBR1 rabbit polyclonal (AB31940, Abcam), and anti-GABA rabbit polyclonal (A2052, Sigma). Fixed cells were then incubated at room temperature in the dark with secondary antibodies conjugated to Alexa Fluor 555 or 488 (Molecular Probes, Thermo Fisher) for 1 h, followed by 1 μg/ml 4’,6-diamidino-2-phenylindole (DAPI, Sigma Aldrich) for 10 min. Imaging was performed using an FV1000 Inverted Confocal System.

### Immunoblotting

FMRP levels were measured in control (HEL46.11) and FXS iPSCs (HEL100.2) and in the NPCs derived from the H1 and FXS hESC lines. Cells were lysed with RIPA buffer (Sigma Aldrich) containing Halt™ Protease and Phosphatase Inhibitor Cocktail (100X) (ThermoFisher, 78,440) for 15 min on ice. The extracts were centrifuged at 15,060 rpm for 15 min at 4 °C and the supernatant was collected. Protein concentration was measured using the Bradford assay (BioRad). Samples were denatured at 95 °C for 10 min in NuPAGE™ LDS Sample Buffer (4X) (ThermoFisher, NP0008) and NuPAGE™ Sample Reducing Agent (10X) (ThermoFisher, NP0009). A total of 40 µg of protein per sample was separated on 9% acrylamide gels at 80 V for 30 min followed by 120 V for 1 h, then transferred to TransBlot Turbo™ Nitrocellulose membrane at 25 V for 7 min using the Trans-Blot Turbo™ Transfer System (Biorad). Membranes were incubated with primary antibodies (1:1,000) at 4 °C overnight followed by peroxidase-conjugated secondary antibodies (1:5,000) for two hours at room temperature. Proteins were visualized using the ChemiDoc™ MP Imaging System (Biorad). The following primary antibodies were used: anti-FMRP mouse (834,601, BioLegend) and anti-Calnexin rabbit (C4731, Sigma). IRDye® 680RD goat anti-Rabbit IgG secondary antibody (Li-Cor) and IRDye® 800CW goat anti-Mouse IgG secondary antibody (Li-Cor) were used as secondary antibodies.

### 5-AzadC treatment

Fresh 5-Aza-2’-deoxycytidine (5AzadC) (Sigma-Aldrich, A3656) was added daily to the mTeSR1 medium (STEMCELL Technologies, Cat. #85,850) of FXS (HEL100.2) iPSC cultures for two consecutive days at a final concentration of 1 μM. A 50 mM stock of 5AzadC was prepared in DMSO. Cells were collected in cold 1 × phosphate-buffered saline for RNA or protein extraction to perform RNA-seq and immunoblotting, respectively.

### RNA extraction, sequencing, and analysis

For hPSC-derived NPCs and neurons, previously published RNA-seq data were utilized from our previous study (hESC-derived neurons: n = 4 biological replicates per genotype, iPSC-derived neurons and hESC-derived NPCs: n = 1 biological replicate per genotype). RNA-seq entailed poly(A)-selected, paired-end 150-bp sequencing of approximately 20 million reads per sample using HiSeq4000 (Novogene, Hong Kong). The RNA-seq data have been deposited in the Gene Expression Omnibus (GEO) under the identifiers GSE117248 [12] and GSE307114.

For untreated and 5AzadC-treated FXS (HEL100.2) iPSCs, fresh RNA extractions were performed using the RNeasy Plus Mini kit (Qiagen) according to the manufacturer’s instructions. Sample quality control was performed using the Agilent 2100 Bioanalyzer or the Agilent 4200 Tapestation. RNA-seq was performed on n = 1 biological replicate per condition (untreated HEL100.2 FXS iPSCs, 5AzadC-treated HEL100.2 FXS iPSCs, and control HEL46.11 iPSCs). Library preparation and RNA-sequencing were performed by the School of Biomedical Engineering Sequencing Facility at the University of British Columbia (Vancouver). Samples were prepped following the standard protocol for the Illumina Stranded mRNA prep (Illumina). Sequencing was performed on the Illumina NextSeq2000 with Paired End 59 bp × 59 bp reads. Sequencing data was demultiplexed using Illumina’s BCL Convert. Alignment and quantification were performed using the STAR aligner (v2.5.2b) using the GRCh38 (hg38) human reference genome and gene annotations were derived from GENCODE Release 44 (GRCh38.p14). Sorted and indexed BAM files were generated using SAMtools, and *FMR1* RNA-seq coverage tracks were plotted using SparK v2.6.2 [16]. Coverage tracks shown in figures represent read coverage depth scaled to the maximum read depth per sample pair. Splice junction reads spanning the intron 1 retention event characteristic of *FMR1-217* were identified from BAM files using SAMtools. Junction-spanning reads were extracted from a 50 bp window flanking the exon 1-exon 2 splice junction (chrX:147,912,230–147,921,932), using reads with CIGAR strings containing ‘N’ indicating intron skipping. Intron 1 reads were defined as those fully contained within chrX:147,912,230–147,921,932. The regions studied span the genomic coordinates of the *FMR1-217* transcript (chrX:147,912,123–147,914,451). The RNA-seq data have been deposited in the GEO under accession number GSE307114.

### Statistical analysis

Junction reads spanning the exon 1-exon 2 junction and intron reads within intron 1 were quantified for each sample using SAMtools. Mis-splicing was evaluated by splicing efficiency [junction/(junction + intron)] and intron retention percent spliced in (PSI) [intron/(junction + intron)]. Pairwise differences between H1, KO1, and FXS neurons were evaluated using two-sided Fisher’s exact tests.

## Results

### *FMR1* RNA is expressed and mis-spliced in FXS NPCs and neurons derived from hESCs

**Fig. 1:**
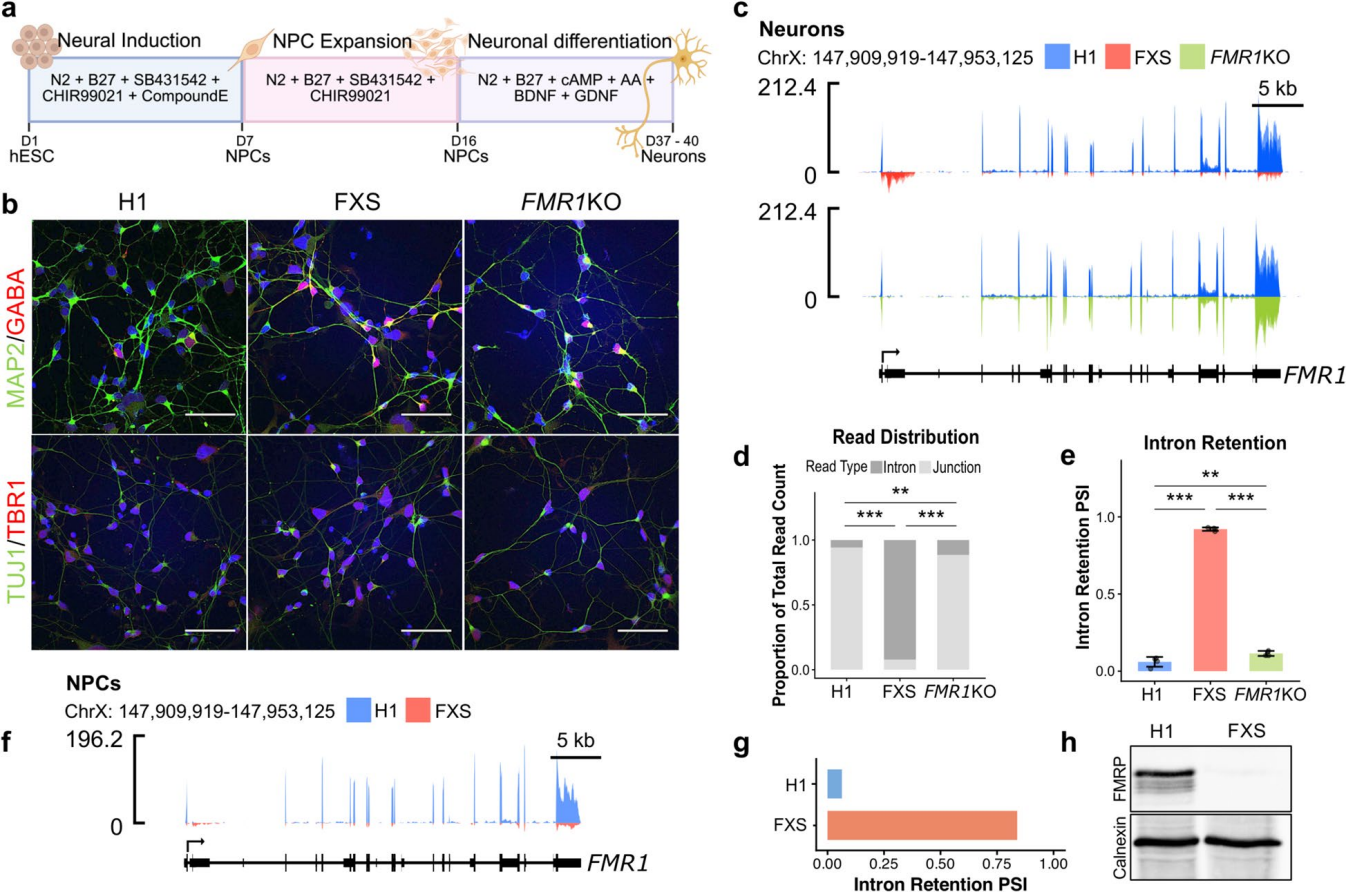
*FMR1-217* detection in NPCs and neurons derived from FXS hESCs. **a** Schematic of NPC and neuronal differentiation workflow. **b** Representative immunofluorescence staining of MAP2/TUJ1-positive GABAergic (GABA +) and glutamatergic (TBR1 +) neurons differentiated from control (H1) and FXS (WCMC-37), and *FMR1*KO hESC lines. Scale bars = 50 μm. **c** RNA-seq coverage tracks across the *FMR1* locus in H1 control (blue), FXS (pink) and *FMR1*KO (green) neurons. **d** Read-type distribution analysis reflecting the proportion of reads mapping to intron 1 (dark grey) or the exon 1-exon 2 junction (light grey) out of the total read count in H1, FXS and *FMR1*KO neurons. **e** Intron retention percent spliced in (PSI). **d**-**e** *N* = 4 per cell line, bars = mean splicing efficiency and error bars = standard deviation. Pairwise differences were evaluated using two-sided Fisher’s exact tests (** *P* < 0.01, *** P < 0.001). **f** RNA-seq coverage tracks across the *FMR1* locus in H1 control (blue), and FXS (pink) hESC derived NPCs. **g** Intron retention PSI in H1 and FXS NPCs (*n* = 1). **h** Immunoblot analysis showing absence of FMRP expression in FXS compared to H1 NPCs. Calnexin serves as the loading control

We used RNA-seq to determine whether *FMR1* mis-splicing occurs in human neurons differentiated from two previously generated human embryonic stem cell (hESC) models of FXS, FXS (WCMC-37) and *FMR1*KO [12, 13] (Fig. 1a). Immunofluorescence staining of the control (WA01, H1), FXS (WCMC-37) and *FMR1*KO neurons 37 days into the differentiation protocol confirmed expression of MAP2 and TUJ1, neuronal markers, with both GABAergic (GABA +) and glutamatergic (TBR1 +) neurons present (Fig. 1b).

The WCMC-37 FXS line was derived from embryos carrying an expanded CGG allele of ~ 450 repeats [12–14]. Despite the presence of a full mutation, FXS (WCMC-37) neurons expressed *FMR1* RNA transcripts, however, at lower levels relative to H1 neurons (Fig. 1c). Importantly, read-type distribution analysis revealed a large enrichment in the proportion of reads mapping to the first intron of *FMR1* as opposed to the exon 1-exon2 splice junction in the FXS compared to the H1 (Fig. 1c,d). The FXS neurons displayed a significant increase in intron retention PSI, and a corresponding significant decrease in splicing efficiency relative to H1 neurons (Fig. 1e, and Supp. Figure 1a, see Additional file 1). Sashimi plots of the *FMR1* locus confirmed split reads supporting the junction between exon 1 and the pseudo-exon within intron 1 in FXS neurons, consistent with the *FMR1-217* isoform described by Shah et al. [10], while H1 neurons showed predominant exon 1-exon 2 junction usage (Supp. Figure 1b, 1c, see Additional file 1).

To investigate whether loss of FMRP contributes to the mis-splicing of its own RNA transcript, we examined RNA-seq data from *FMR1*KO hESC-derived neurons. The *FMR1*KO hESC line was previously generated using CRISPR/Cas9 targeting exon 3 of *FMR1* in the H1 hESC line [12]. This isogenic H1 *FMR1*KO line represents a protein loss-of-function model in which *FMR1* transcription remains intact but with no functional FMRP being produced, which is distinct from the transcriptional silencing observed in methylated FXS full-mutations [12]. While *FMR1* RNA transcripts were detected at levels comparable to the H1 neurons, only a small proportion of the reads in the *FMR1*KO neurons mapped to the first intron (Fig. 1c). The *FMR1*KO neurons showed a significant but modest increase and decrease in intron retention PSI and splicing efficiency, respectively, when compared to the H1 (Fig. 1d, and Supp. Figure 1a, see Additional file 1). Sashimi plots confirmed that *FMR1*KO neurons retained predominantly canonical exon 1-exon 2 splicing, in contrast to FXS neurons which showed abundant split reads to the intron 1 pseudo-exon characteristic of *FMR1-217* (Supp. Figure 1b, 1c, see Additional file 1). Therefore, FMRP loss alone, in the absence of an expanded CGG tract, causes only a modest increase in mis-splicing of its own RNA transcript and minimal formation of *FMR1-217*. *FMR1*KO RNA-seq analysis was performed in neurons; as this line carries a normal CGG repeat tract, the putative determinant of *FMR1-217* formation, a similar pattern would be expected in *FMR1*KO NPCs.

Given the pronounced enrichment of *FMR1*-217 observed in FXS neurons, we next examined whether this mis-spliced isoform could be detected earlier during neural development in NPCs generated using the same differentiation protocol (Fig. 1a). Consistent with our findings in the neurons, FXS NPCs displayed an increase in intron retention PSI and decrease in splicing efficiency (Fig. 1f, g, and Supp. Figure 1 d, see Additional file 1), and sashimi plots confirmed split reads consistent with the *FMR1-217* isoform (Supp. Figure 1e, see Additional file 1). The more pronounced intronic reads (corresponding to *FMR1-217*) in FXS neurons compared to NPCs could reflect several factors: (1) higher overall *FMR1* transcription in neurons compared to NPCs, resulting in proportionally more mis-spliced transcripts; (2) developmental stage-dependent changes in the splicing machinery that may affect the efficiency of mis-splicing in more mature cell types; or (3) differential stability of the *FMR1-217* transcript in neurons versus NPCs.

Despite *FMR1* expression at the RNA level, FXS NPCs exhibited complete loss of FMRP, as confirmed by immunoblotting (Fig. 1h). Immunoblotting was performed in NPCs, which can be generated more rapidly and in sufficient quantities for protein-level analyses compared to neuronal cultures. The absence of FMRP in FXS neurons differentiated from the same WCMC-37 line has been previously confirmed by immunoblotting and proteomics [17].

### Reactivation of expanded *FMR1* in FXS iPSCs produces mis-spliced transcripts and fails to restore FMRP expression

**Fig. 2:**
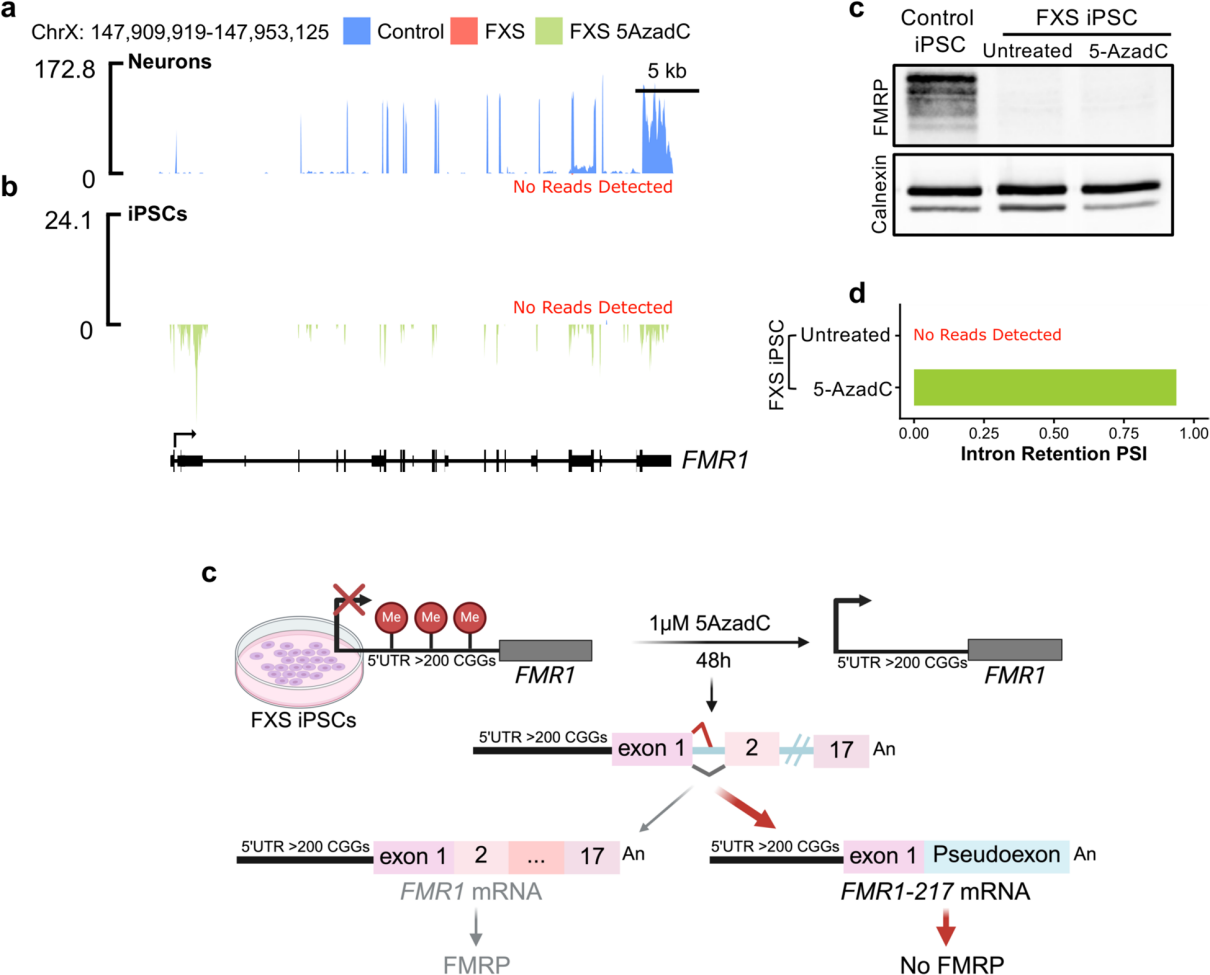
Reactivation of *FMR1* in FXS iPSCs produces mis-spliced transcripts and fails to restore FMRP expression. **a**-**b** RNA-seq coverage tracks across the *FMR1* locus in control (blue) and FXS untreated (pink) iPSC derived neurons (**a**) and FXS untreated (pink) and 5AzadC-treated (green) iPSCs, 1 μM for 48 h (**b**). FXS untreated neurons and iPSCs show complete transcriptional silencing of *FMR1*, indicated by the absence of RNA reads. **c** Western blot analysis showing absence of FMRP in both untreated and 5AzadC-treated FXS iPSCs despite successful transcriptional reactivation of the *FMR1* locus. Calnexin serves as loading control. **d** Intron retention percent spliced in FXS 5AzadC treated iPSCs (*n* = 1). **e** Schematic illustrating the effects of *FMR1* reactivation using 5AzadC treatment in the presence of an expanded CGG repeat tract. While the treatment leads to the successful transcriptional reactivation of *FMR1*, enrichment in mis-splicing and retention of the first intron of *FMR1* is observed. This results in the formation of an aberrantly spliced isoform corresponding to *FMR1*-217, which is predicted to code for a truncated nonfunctional protein. Therefore, *FMR1* mis-splicing seems to partly underlie the absence of FMRP in patients with partially methylated full mutations showing residual *FMR1* RNA expression

We next investigated *FMR1* mis-splicing using previously described control (HEL46.11) and FXS (HEL100.2) patient-derived iPSCs [15]. The HEL100.2 FXS iPSC line carries a fully methylated full mutation (> 200 CGG repeats). Unlike the WCMC-37 FXS line, which exhibits residual *FMR1* transcription (Fig. 1c), the HEL100.2 FXS iPSC-derived neurons show complete transcriptional silencing when compared to control (Fig. 2a). This makes the HEL100.2 FXS iPSCs suitable for assessing the effects of pharmacological reactivation.

To reactivate the silenced *FMR1* gene, we treated the HEL100.2 FXS iPSCs with the DNA methylation inhibitor 5-AzadC for 48 h [18–20]. 5AzadC administration successfully reactivated *FMR1* transcription as indicated by RNA reads detected in the treated but not untreated FXS iPSCs (Fig. 2b). However, it failed to restore FMRP expression (Fig. 2c). The lack of FMRP production in the 5AzadC-treated FXS cells is consistent with *FMR1* mis-splicing, as RNA-seq revealed an enrichment in intron 1 retention PSI and a corresponding decrease in splicing efficiency (Fig. 2b, d, and Supp. Figure 2a, see Additional file 1). Sashimi plots confirmed splice junction usage consistent with the *FMR1-217* isoform in 5-AzadC-treated FXS iPSCs but not in untreated cells or controls (Supp. Figure 2b, 2c, see Additional file 1).

## Discussion

Our findings provide independent support for the recent observation that expanded *FMR1* transcripts undergo mis-splicing, which may contribute to the reduction or absence of FMRP in FXS [10]. A mis-spliced *FMR1* isoform, *FMR1-217*, has recently been reported in leukocytes, lung-derived fibroblasts and postmortem cortex samples from individuals with FXS [10]. Our study extends these findings to human NPCs and neurons differentiated from FXS hESCs, demonstrating that this phenomenon occurs in disease-relevant neural cell types.

Our data suggest that *FMR1* RNA undergoes mis-splicing in the presence of an expanded CGG tract. Additionally, while several studies have shown widespread splicing dysregulation in the absence of FMRP, our findings using *FMR1*KO cells demonstrate that FMRP loss alone, in the context of a normal CGG repeat tract, contributes only modestly to the splicing defect and *FMR1***-**217 formation [2, 10, 21, 22]. Whether FMRP loss has a more pronounced contributory role in mis-splicing in the setting of expanded repeats remains to be determined. Importantly, the CRISPR/Cas9 edit employed in this work targets exon 3, which is downstream of the intron 1 region involved in *FMR1-217* formation, and the *FMR1*KO line shows normal FMR1 splicing, confirming that the exon 3 edit did not disrupt splicing regulatory elements relevant to intron 1 processing.

The absence of FMRP in FXS NPCs expressing *FMR1* RNA is unlikely to result solely from the lower levels of *FMR1* RNA observed, as previous studies have shown complete FMRP loss in FXS patients expressing *FMR1* RNA at levels comparable to typically developing individuals [7, 9]. Instead, FMRP reduction or loss may result from multiple converging mechanisms, including mis-splicing of *FMR1* RNA in the presence of an expanded CGG tract [10]. The *FMR1-217* isoform likely forms a truncated polypeptide with unknown function, if any, preventing the production of functional FMRP [10].

An important consideration is that expanded CGG-containing *FMR1* transcripts may serve as templates for non-AUG RAN translation, producing polyglycine-containing proteins (FMRpolyG) implicated in FXTAS pathogenesis [23–25]. However, most individuals with FXS full mutations do not develop FXTAS-like features or accumulate significant intranuclear inclusions. While rare inclusions (0.1–1.3% of cells) have been reported in the brains of older males with FXS, this is far below levels observed in FXTAS [26]. This likely reflects the fact that in most FXS individuals with fully methylated full mutations, *FMR1* transcription is effectively silenced; in cases where some transcription occurs, levels may be below the threshold for significant RAN protein accumulation.

The molecular mechanisms by which expanded CGG repeats induce mis-splicing remain to be elucidated, but several non-mutually exclusive possibilities exist. Expanded CGG/CCG repeats form stable R-loop structures that could interfere with co-transcriptional splicing [27–29]. Expanded CGG repeats in the nascent transcript may form stable secondary structures such as hairpins and G-quadruplexes that could impair spliceosome assembly [7, 8, 30]. Chromatin modifications associated with the expanded CGG tract may affect splice site recognition [31, 32]. The formation of *FMR1-217* as a 1.8 kb transcript suggests that premature transcription termination and polyadenylation, as has been shown for other expanded repeats [33], within intron 1 may also contribute to FMRP absence. The detection of *FMR1-217* in poly(A)-selected libraries supports this possibility.

*FMR1* mis-splicing in the presence of expanded CGG repeats may pose challenges for therapeutic approaches aimed at reactivating *FMR1* in FXS patients. Not only would *FMR1* mis-splicing impair FMRP production upon *FMR1* activation, but the formation of aberrant CGG expansion-containing isoforms, such as *FMR1-217,* may trigger gain-of-function RNA toxicity [27, 34, 35]. These findings are consistent with the notion that therapeutic approaches designed to reactivate the expanded *FMR1* locus will need to simultaneously address *FMR1* mis-splicing to achieve meaningful restoration of FMRP production and clinical outcomes. This includes the ASO-mediated rescue of FMRP production through blocking *FMR1-217* formation, as demonstrated by Shah et al. [10]. However, the rescue was performed in lymphoblastoid cell lines, and whether a similar strategy can effectively rescue FMRP in post-mitotic neurons remains to be determined and represents a critical future direction.

Human pluripotent stem cell lines represent clonal or near-clonal populations with more uniform CGG repeat and methylation profiles compared to patient tissues, which may explain the complete absence of FMRP we observe versus the residual expression reported in 12–41% of males with FXS [3]. Previous studies showed partial FMRP restoration following 5AzadC treatment in lymphoblastoid cell lines [18, 19], but those cells differ from iPSCs in epigenetic landscape and proliferative properties. Our data are also consistent with mis-spliced transcripts arising from demethylated, transcriptionally active loci.

We acknowledge that short-read RNA-seq cannot resolve the full diversity of *FMR1* transcript isoforms. The presence of reads across all *FMR1* exons could reflect co-expression of *FMR1-217* and canonical full-length transcripts, or the existence of longer aberrant isoforms. Long-read sequencing will be essential to characterize the spectrum of transcripts from expanded *FMR1* alleles. The absence of FMRP likely reflects the combined effects of multiple mechanisms, including mis-splicing, translational impairment by the expanded CGG tract [9], and potentially other forms of aberrant RNA processing.

### Limitations

This study provides important insights into *FMR1* mis-splicing in FXS; however, several limitations should be noted. The use of a limited number of FXS hESC and iPSC lines restricts the generalizability of our findings across the genetic and epigenetic diversity observed in FXS patients. Furthermore, although our study establishes a correlation between CGG expansion and *FMR1* mis-splicing, the underlying molecular mechanisms remain unresolved, with no direct investigation of splicing regulators or RNA structural alterations. Finally, the absence of functional assays limits our assessment of the pathogenic relevance of the mis-spliced *FMR1-217* isoform, particularly with regard to its potential toxicity or RNA-mediated effects. Additionally, we did not perform RT-PCR-based validation of *FMR1-217* in our neural cell models, although this isoform has been validated by RT-PCR and sequencing in other cell types by Shah et al. [10]. The limited number of biological replicates, particularly for the 5AzadC treatment experiments (n = 1), and the use of single FXS lines per cell type represent additional limitations. Furthermore, our *FMR1*KO experiment demonstrates that FMRP loss alone causes modest mis-splicing in the absence of expanded CGG repeats. One approach to more directly test the role of FMRP deficiency in the generation of *FMR1-217* would be to express ectopic FMRP (from FMR1 cDNA for example) in cells harbouring a transcriptionally active expanded CGG tract, such as the FXS hESC line and assess whether this rescues the mis-splicing and *FMR1*−217 enrichment observed. Finally, we did not re-characterize CGG repeat sizes and methylation status at the time of our experiments, which is relevant given the inherent instability of CGG repeats during cell culture.

## Conclusions

Our analyses reveal that while *FMR1* is transcribed in NPCs and neurons derived from FXS hESCs with an expanded CGG tract, no FMRP is produced, likely due in part to mis-splicing and potentially other mechanisms including translational impairment by the expanded CGG tract. Furthermore, treatment of FXS patient-derived iPSCs with 5AzadC for 48 h reactivated the *FMR1* locus but failed to restore FMRP expression, instead producing high levels of the *FMR1-217* isoform. These findings build on the key results from Shah and colleagues and have significant implications for understanding FXS pathogenesis and developing effective therapeutic strategies. The observation that mis-splicing persists even after pharmacological reactivation suggests that successful FXS therapies targeting the expanded *FMR1* locus must address both transcriptional silencing and aberrant splicing mechanisms.

## Supplementary Information

Additional file 1. Supplementary figures.

## Data Availability

All data generated or analyzed during this study have been deposited in the GEO (GSE117248 and GSE307114). Any additional information related to the current study is available from the corresponding author on reasonable request.

## Abbreviations

5AzadC: 5-Aza-2’-deoxycytidine
*FMR1*: Fragile X Messenger Ribonucleoprotein 1 (gene)
*FMR1-217*: Mis-spliced FMR1 isoform retaining intron 1
FMRP: Fragile X Messenger Ribonucleoprotein
FXS: Fragile X Syndrome
FXPOI: Fragile X-associated Primary Ovarian Insufficiency
FXTAS: Fragile X-associated Tremor/Ataxia Syndrome
hESC: Human Embryonic Stem Cell
iPSC: Induced Pluripotent Stem Cell
KO: Knockout
MAP2: Microtubule-Associated Protein 2
NPC: Neural Progenitor Cell
TDI: Typically Developing Individual
UTR: Untranslated Region
