# Supplementary material for: Mis-spliced FMR1 transcripts in human fragile X syndrome neural progenitors and neurons
Source: J Neurodev Disord. 2026 Apr 2;18:26. doi: 10.1186/s11689-026-09686-0 (PMC13169608; doi:10.1186/s11689-026-09686-0)
Supplement: Supplementary file 1 — Additional file 1. Supplementary figures. [file 11689_2026_9686_MOESM1_ESM.pdf]

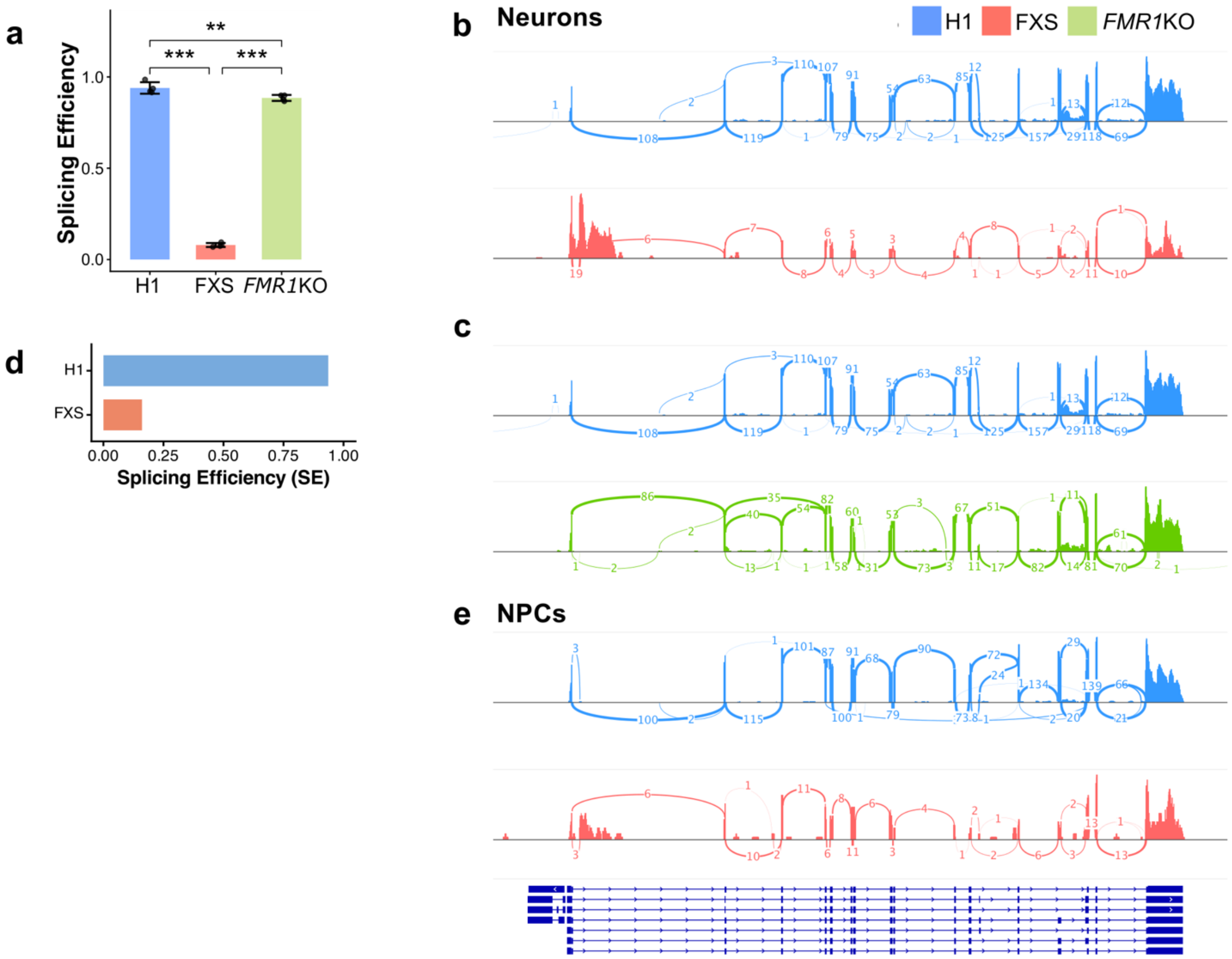

Supplementary Fig 1. Altered splice junction usage and reduced splicing efficiency in FXS compared to H1. (a) Splicing efficiency [junction / (junction + intron)] performed on control H1, FXS (WCMC-37) and FMR1KO hESC derived neurons. N=4 per cell line, bars = mean splicing efficiency and error bars = standard deviation. Pairwise differences were evaluated using two-sided Fisher's exact tests (** P < 0.01, *** P < 0.001). **(b-c)** Sashimi plot showing RNA-seq coverage and splice junction usage across *FMR1* locus in H1, FXS (WCMC-37) and *FMR1*KO neurons (n=4). Genomic coordinates are plotted on x-axis and read density on y-axis. **(d)** Splicing efficiency performed on control H1, FXS (WCMC-37) hESC derived NPCs (n=1). **(e)** Sashimi plot of the *FMR1* locus in H1 and FXS (WCMC-37) NPCs (n=1). Genomic coordinates are plotted on x-axis and read density on y-axis.

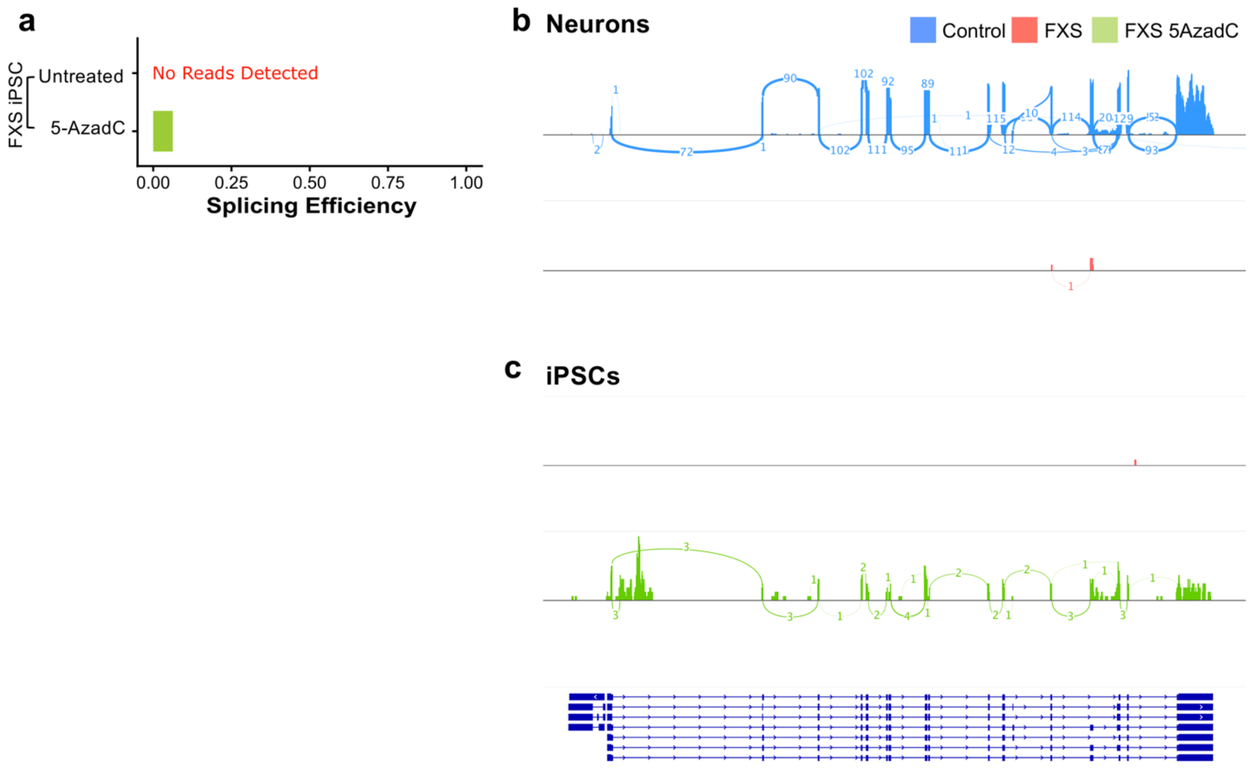

Supplementary Fig 2. Altered splice junction usage and low splicing efficiency upon 5AzadC reactivation of the transcriptionally silent *FMR1* locus in FXS iPSCs. (a) Splicing efficiency [junction / (junction + intron)] performed on control and HEL100.2 FXS iPSCs (n=1). **(b-c)** Sashimi plot showing RNA-seq coverage and splice junction usage across *FMR1* locus in control and HEL100.2 FXS iPSCs-derived neurons **(b)** and iPSCs **(c)** (n=1). Genomic coordinates are plotted on x-axis and read density on y-axis.
